# How Angioedema Quality of Life Questionnaire Can Help Physicians in Treating C1-Inhibitor Deficiency Patients?

**DOI:** 10.1007/s12016-021-08850-9

**Published:** 2021-03-03

**Authors:** Zsuzsanna Balla, Bettina Ignácz, Lilian Varga, Kinga Viktória Kőhalmi, Henriette Farkas

**Affiliations:** 1 Hungarian Angioedema Center of Reference and Excellence, Department of Internal Medicine and Haematology, Semmelweis University, Budapest, Hungary; 2 School of PhD Studies, Semmelweis University, Budapest, Hungary; 3 Department of Laboratory Medicine, Semmelweis University, Budapest, Hungary; 4 Department of Internal Medicine and Haematology, Semmelweis University, Budapest, Hungary; 5 Department of Rheumatology and Clinical Immunology, Semmelweis University, Budapest, Hungary; 6 Department of Rheumatology, Hospital of the Hospitaller Brothers of Saint John of God, Budapest, Hungary

**Keywords:** Acquired angioedema, Questionnaire, C1-inhibitor deficiency, Complement, Hereditary angioedema, Quality of life

## Abstract

The Angioedema Quality of Life Questionnaire (AE-QoL) is an angioedema (AE)-specific validated questionnaire, which surveys the quality of life of diagnosed patients. The questionnaire has been used in multiple clinical trials. Our aim was to investigate how the questionnaire can assist physicians in the everyday practice of following up and managing C1-inhibitor deficiency patients. In a prospective trial conducted in our center between 2016 and 2018, 125 hereditary angioedema and 10 diagnosed with acquired angioedema completed an AE-QoL during their annual follow-up visit. Laboratory indices (i.e., complement levels) were obtained for each patient. Statistical analysis comparing clinical data with QoL parameters was performed. Results of the analysis show that AE-QoL total score and number of AE attacks per year correlated well (*r* = 0.47; *p* < 0.0001). Women reached higher AE-QoL total score values than men, over a 3-year period (*p* = 0.0014). The highest AE-QoL total scores were reached by the 41–60-year age group; we obtained a similar result, when analyzing the four domains. No correlation was found between the AE-QoL total score and complement parameters. Patients with a negative correlation between AE-QoL total score and number of AE attacks had a positive correlation with psychologic attributes like fatigue/mood and fears/shame domains. Patients that acquired HAE showed a significant correlation between the annual number of AE attacks and the AE-QoL total scores (*r* = 0.46; *p* < 0.0001). The study establishes the use of AE-QoL as a clinical tool for follow-up which can help in the complex assessment of both hereditary and acquired HAE patients, and help to develop better therapeutic strategies.

## Introduction

Angioedema (AE) with C1-inhibitor deficiency (C1-INH-AE) belongs to the group of bradykinin (BK)-mediated AEs. These AEs are rare, potentially life-threatening, and characterized by recurrent angioedema episodes [1, 2]. They can be further divided into hereditary and acquired AEs. Hereditary AE with C1-INH deficiency (C1-INH-HAE) is caused by mutations in the *SERPING1* gene, with an autosomal dominant inheritance pattern [3, 4].

Acquired AE with C1-INH deficiency (C1-INH-AAE) is mostly developed due to an underlying disease (mostly lymphomas) where a consumption of the C1-INH protein and/or formation of anti-C1-INH autoantibodies cause decreased C1-INH quantity and function [5]. Clinical manifestations of C1-INH-AE might be easily misdiagnosed, resulting in a diagnosis delay of years, or even decades [6, 7].

 Recurrent AE attacks are non-pruritic and non-pitting swelling of the subcutaneous and/or submucosal tissues [8]. The subcutaneous edemas typically occur on the face, neck, extremities, trunk, or genitalia. A non-pruritic, red, map-like, highly characteristic serpiginous rash (erythema marginatum) can occur as a prodromal symptom [7]. Gastrointestinal AE attacks showing clinical symptoms similar to acute abdomen often result in unnecessary surgical interventions [9]. Moreover, the delay of diagnosis can have fatal consequences; if the patient develops laryngeal edema, which causes the obstruction of the upper airways the use of conventional treatments (e.g., antihistamines, corticosteroids, adrenaline), suffocation may ensue, unless a specific therapy is used [10–12].

The health-related quality of life (HR-QoL) of C1-INH-HAE patients is significantly impacted by the fact that the severity and frequency of AE attacks are unpredictable and shows intra- and inter-individual differences [13]. Depending on the extent and localization, AEs can be painful and therefore influence the physical and mental health of the patient. AE attacks restrict patients in performing their everyday routine, and patients may miss work or school unexpectedly and frequently; AEs negatively affect working effectivity, learning, social life, and relationships. Patients suffering from recurrent AEs are often stigmatized as unreliable employees; moreover, patients are continuously under stress from the possibility of the reoccurrence of new attacks and C1-INH-HAE patients develop depression and anxiety more frequently [14, 15]. Therefore, it can be concluded that AE has an important effect on the HR-QoL, which should be considered when evaluating the general burden of the disease when developing a treatment strategy. Several generic questionnaires are available to assess HR-QoL. Previously, general and specific dermatological questionnaires were used to measure the QoL of C1-INH-HAE patients, such as the most often used Short Form 12, Short Form 36 Health Survey, EuroQol 5, or the Dermatology Life Quality Index. The advantage of these questionnaires is that they are useful for different diseases. Notwithstanding, generic questionnaires also have limitations, since their specificity and sensitivity for a specific diseases are low. The disease-specific questionnaire is required to compare different therapeutic results of certain diseases. Indeed, several HAE-specific HR-QoL questionnaires have been developed to measure the health status of C1-INH-HAE patients. As of today, 3 questionnaires are available that are specific to angioedema [16, 17].

The Angioedema Quality of Life Questionnaire (AE-QoL) consists of 17 items, divided into 4 domains: functioning, fatigue/mood, fears/shame, and food. These domains are defined for the 4 weeks preceding the time of filling of the questionnaires. The instrument development involved patients with different types of AE (chronic spontaneous urticaria, C1-INH-HAE, idiopathic AE), allowing to compare different types of angioedema. These questionnaires have been widely used in clinical trials and practice since its development [18, 19]. In 2016, Weller et al. proved a correlation between the AE-QoL total score and number of AE attacks occurring 4 weeks preceding the completion of the questionnaire. A change of six points out of the AE-QoL total score was regarded as a significant change for the patient [20]. The Hereditary Angioedema Quality of Life (HAE-QoL) questionnaire proposed by Prior and Caballero et al. is the first disease-specific questionnaire, adopted for HAE with C1-INH deficiency (C1-INH-HAE) [21, 22].

Additionally, the Angioedema Activity Score (AAS) is the first instrument to measure disease activity. It is a symptom-specific, subjective questionnaire, developed for the clinical follow-up of AEs. This questionnaire was validated and can apply for all the recurrent AEs, including C1-INH-HAE. Using it in parallel with the Urticaria Activity Score (UAS), it is useful for surveying chronic spontaneous urticaria patients with angioedema symptoms [23].

The primary purpose of our research was to use the AE-QoL in the assessment of the HR-QoL of patients with C1-INH deficiency and diagnosis of either hereditary or acquired angioedema. We sought to compare the HR-QoL data with clinical data distinctive for the disease, including complement laboratory parameters recognized as biomarkers of disease activity. In 2011, our team demonstrated that the severity of the disease can be predicted by the level of functional C1-INH and C1rC1sC1-INH complex [24]. Since the severity of the disease is well shown by the AE-QoL total score, we wanted to observe if there is a correlation between the AE-QoL total score and the complement parameters.

## Methods

### Patients

Altogether, out of 197 C1-INH-HAE patients treated in HACRE, 125 adult patients participated in the study, which consisted 72 women (aged 18–86, median 41.5 years) and 53 men (aged 18–76, median 43 years). In 2018, 10 C1-INH-AAE patients were also enrolled in the study. The study protocol was approved by the institutional review board of Semmelweis University of Budapest, and informed consent was obtained from the participants in accordance with the Declaration of Helsinki.

### Data Collection

C1-INH deficiency patients treated in the Hungarian Angioedema Center of Reference and Excellence (HACRE) had a follow-up visit at least once a year, at which we recorded the number of AE attacks that occurred in the past year; the body location of the AE attack, based on the data recorded in patient diaries; discharge reports; and other medical records. In addition, complement levels were determined from blood samples (total classic complement cascade, C3, C4, C1-INH concentration level, and C1-INH functional activity). Data of AE symptoms and complement parameters were recorded in the National Angioedema Register. C1-INH-HAE patients who participated in the prospective trial conducted in 2016–2018 have completed the AE-QoL questionnaire at their annual follow-up visit. For C1-INH-AAE patients, the questionnaire was introduced in 2018; due to this, their data is only available for that year.

### Structure of the AE-QoL Questionnaires

The following answers could be given to the questions within each domain, which were scored from 1 to 5: 1 = never, 2 = rarely, 3 = sometimes, 4 = often, 5 = very often [20].

For the evaluation of the questionnaire, the total score was measured based on 17 questions, and the domains were evaluated separately. The Functioning domain contains 4, the Fatigue/Mood domain contains 5, the Fears/Shame domain contains 6, and the Food domain contains 2 questions. The AE-QoL domain scores and total scores were counted with the following formula: (total score of a domain − the number of questions in that domain) / (maximum score of the domain − the minimum score of that domain) × 100. The AE-QoL domain scores corresponded with the average of items found in one domain. The scores were given in percentages. Thus, both the domain scores, both the total score were between 0 and 100. A higher score means worse, while a lower score means better QoL [18].

After evaluating the questionnaire, the results were compared with the gender, age, number of annual AE attacks and its localization, and complement laboratory parameters.

### Statistical Analysis

After the evaluation of the AE-QoLs, a statistical analysis was performed with GraphPad Prism 5.0 (GraphPad Software, San Diego, CA, USA). Differences between the results of the AE-QoL total and domains scores per year were measured with a paired *t*-test. Mann-Whitney assay was used to evaluate the difference between the different groups (gender, age), Spearman correlation was used to evaluate the correlation between AE-QoL total score and the number of AE attacks per year, localization, and complement levels. Only *p* < 0.05 values were considered statistically significant.

## Results

### C1-INH-HAE Patients

A total of 125 C1-INH-HAE adult patients (53 men, 72 women, average age 42 years range 18–86 years) enrolled in the study and completed the AE-QoL questionnaire in at least one of the study years. Sixty-one patients completed the questionnaires all 3 successive years, 39 patients completed 2 successive years, and 1 year’s data is available from 25 patients.

#### Evaluation of the AE-QoL Total Score

The median AE-QoL total score (25th and 75th percentiles) in 3 years (2016–2018) is 20.6 (5.9; 36.8). This value for 2016 was 20.6 (2.9; 33.8), for 2017 22.1 (6.6; 36.8), and 18.4 for 2018 (5.9; 38.6). Statistically, there was no significant difference between AE-QoL total scores of the observation years, and no outlier was observed. When evaluating the data of 61 patients who completed the questionnaires in 3 successive years, no statistical difference was found (Table 1)*.*

**Table 1:** Three-year breakdown of AE-QoL total scores

	2016–2018	2016	2017	2018
AE-QoL total score	20.6 (5.9–36.8) (*n* = 286)	20.6 (2.9–33.8) (*n* = 95)	22.1 (6.6–36.8) (*n* = 97)	18.4 (5.9–38.6) (*n* = 94)
Distribution of AE-QoL total score based on gender				
Women	26.5 (7.4–41.2) (*n* = 165)	23.5 (3.3–36.4) (*n* = 56)	28.7 (11–41.2) (*n* = 54)	23.5 (7.4–44.1) (*n* = 55)
Men	13.2 (2.9–28.7) (*n* = 121)	17.7 (2.9–30.9) (*n* = 39)	13.2 (4.4–26.5) (*n* = 43)	11.8 (2.9–26.5) (*n* = 39)
Distribution of AE-QoL total score based on age groups				
18–40 years	12.5 (0; 37.5) (*n* = 141)	20,6 (8.1; 33.8) (*n* = 50)	20.6 (8.8; 38.2) (*n* = 47)	17.7 (6.3; 40.1) (*n* = 44)
41–60 years	23,5 (5.9; 38.2) (*n* = 105)	27.9 (2.9; 38.2) (*n* = 31)	23.5 (6.6; 35.3) (*n* = 37)	19.1 (6.6; 40.4) (*n* = 37)
Over 60 years	11.9 (0; 27.9) (*n* = 40)	8.8 (0; 19.1) (*n* = 14)	22.1 (0.7; 28.7) (*n* = 13)	14.7 (0.7; 27.9) (*n* = 13)
Distribution of AE-QoL total scores based on age groups and gender				
Women (18–40)	27.9 (8.1; 45.6) (*n* = 85)	25 (5.5; 45.6) (*n* = 32)	33.8 (7.4; 50) (*n* = 27)	28.7 (8.1; 44.9) (*n* = 26)
Women (41–60)	27.9 (11.8; 41.5) (*n* = 54)	27.9 (5.9; 35.3) (*n* = 15)	30.9 (14; 46.3) (*n* = 18)	27.9 (9.6; 46.3) (*n* = 21)
Women (60 <)	15.4 (0; 27.9) (*n* = 26)	8.8 (0; 22.1) (*n* = 9)	22.1 (0; 27.9) (*n* = 9)	15.4 (0; 27.2) (*n* = 8)
Men (18–40)	11.8 (7.2; 20.6) (*n* = 56)	13.2 (8.1; 22.8) (*n* = 18)	12.5 (8.; 20.2) (*n* = 20)	11 (5.5; 25) (*n* = 18)
Men (41–60)	17.7 (0; 38.2) (*n* = 51)	27.2 (0.7; 40.4) (*n* = 16)	16.2 (1.5–32.4) (*n* = 19)	15.4 (0; 37.5) (*n* = 16)
Men (60 <)	7.4 (1.5; 30.5) (*n* = 14)	10.3 (0; 23.5) (*n* = 5)	16.9 (2.2; 47.1) (*n* = 4)	2.9 (1.5; 34.6) (*n* = 5)

Distribution of AE-QoL total scores per year; based on gender, age groups and age groups in genders. The first number is the median, and numbers in brackets are the 25th and 75th percentile values, respectively

*AE-QoL* Angioedema Quality of Life Questionnaire

##### Distribution of the AE-QoL Total Score Based on Gender

The AE-QoL total score of women and men were compared for every year. In 2017, there were a significant difference (*p* = 0.0039) between the two groups, namely, women had a higher score. This tendency was present in the other two years as well (Table 1).

##### Distribution of AE-QoL Total Scores Based on Age Groups

Next, the population was divided based on age groups: the 1st group included patients aged 18–40 years, the 2nd group consisted of patients between 41 and 60 years, and the 3rd group consisted of patients over 60 years of age. The second group received the highest AE-QoL score. Significant differences were found between the AE-QoL total scores of the 1st and 3rd and the 2nd and 3rd age groups (*p* = 0.0104, *p* = 0.0035), respectively). This tendency can be also observed in the annual breakdown (Table 1).

##### Distribution of AE-QoL Total Scores Based on Age Groups and Gender

Age groups were broken down by gender and age in the following method: we compared the different age groups, divided by gender for each year, and also compared the women and men in the same age group in a certain year.

Women compared with men reached a higher score in every age group (except in 2016 in the group over the age of 60); in 2017, there was a significant difference between two age groups (in the group of 18–40 *p* = 0.0330; in the group of 41–60 *p* = 0.0466), and this tendency was observed for all the other years. Women over 60 reached a significantly lower total AE-QoL score than women in the first and the second age group; this tendency was not observed in men (Table 1).

#### Evaluating Four AE-QoL Domains

Annual changes of the four domains (Function, Fatigue/Mood, Fears/Shame, Food) were also evaluated, but no difference was found over the years. The highest score was reached in the Function and Fatigue/Mood domains (Table 2).

**Table 2:** Distribution of AE-QoL domain scores in three successive annual visits

		Function	Fatigue/Mood	Fears/Shame	Food
2016–2018 (*n* = 286)		18.8 (0; 43.8)	20 (5; 40)	16.7 (0; 33.3)	12.5 (0; 37.5)
2016 (*n* = 95)		18.8 (0; 43.8)	20 (0; 40)	16.7 (0; 33.3)	0 (0; 25)
2017 (*n* = 97)		25 (0; 43.8)	20 (5; 40)	16.7 (8.3;37.5)	12.5 (0; 31.3)
2018 (*n* = 94)		18.8 (0; 43.8)	20 (3.8; 40)	16.7 (0; 33.3)	12.5 (0; 37.5)
P1, P2, P3		ns., ns., ns	ns., ns., ns	ns., ns., ns	ns., ns., ns
Scores of domains by gender					
2016–2018	Women (*n* = 165)	25 (0; 43.8)	25 (5; 40)	20.8 (4.2; 41.7)	12.5 (0; 50)
	Men (*n* = 121)	12.5 (0; 31.3)	15 (5; 32.5)	12.5 (0; 25)	0 (0; 25)
	*p* value	0.0032	ns	0.0021	0.004
2016	Women (*n* = 56)	15.6 (0; 43.8)	17.5 (0; 40)	16.7 (0; 33.3)	0 (0; 37.5)
	Men (*n* = 39)	18.8 (0; 25)	20 (5; 40)	12.5 (0; 29.2)	0 (0; 25)
	*p* value	ns	ns	ns	ns
2017	Women (*n* = 54)	31.3 (4.7; 43.8)	25 (10; 45)	25 (8.3; 41.7)	25 (0; 50)
	Men (*n* = 43)	12.5 (0; 31.3)	15 (5; 30)	12.5 (4.2; 25)	12.5 (0; 25)
	*p* value	0.0113	0.0403	0.0109	0.0101
2018	Women (*n* = 55)	18.8 (0; 50)	20 (5; 45)	20.8 (4.2; 50)	12.5 (0; 50)
	Men (*n* = 39)	12.5 (0; 31.3)	10 (0; 30)	12.5 (0; 20.8)	0 (0; 25)
	*p* value	ns	ns	ns	ns
Scores of domains by age groups					
2016–2018	18–40 years (*n* = 141)	18.8 (0; 43.8)	15 (5; 37.5)	16.7 (4.2; 41.7)	12.5 (0; 37.5)
	41–60 years (*n* = 105)	25 (0; 43.8)	25 (2.5; 42.5)	16.7 (4.2; 35.4)	25 (0; 37.5)
	over 60 years (*n* = 40)	0 (0–23.4)	15 (0–48.8)	8.3 (0–25)	0 (0; 25)
	P1*, P2*, P3*	ns., 0.0002, 0.002	ns.,ns., ns	ns., 0.0044, 0.0201	ns., 0.0093, 0.0006
2016	18–40 years (*n* = 50)	18.8 (0; 43.8)	15 (5; 40)	18.8 (0, 36.5)	0 (0; 37.5)
	41–60 years (*n* = 31)	31.3 (0; 43.8)	25 (0, 45)	25 (0;33.3)	25 (0; 37.5)
	over 60 years (*n* = 14)	0 (0; 1.6)	10 (0; 35)	4.2 (0; 17.7)	0 (0; 0)
	P1*, P2*, P3*	ns., 0.0004, 0.0024	ns., ns., ns	ns., ns., ns	ns., 0.0234, 0.0037
2017	18–40 years (*n* = 47)	18.8 (0; 43.8)	20 (10; 40)	16.7 (8.3; 41.7)	12.5 (0; 50)
	41–60 years (*n* = 37)	31.3 (0; 43.8)	20 (5; 37.5)	20.8 (8.3; 37.5)	25 (0; 31.3)
	over 60 years (*n* = 13)	6.3 (0; 40.6)	15 (2.5; 50)	12.5 (0; 35.4)	0 (0; 25)
	P1*, P2*, P3*	ns., ns., ns	ns., ns., ns	ns., ns., ns	ns., ns., ns
2018	18–40 years (*n* = 44)	18.8 (1.6; 43.8)	15 (5; 25)	18.8 (4.2; 50)	12.5 (0; 37.5)
	41–60 years (*n* = 37)	25 (0; 50)	25 (0; 57.5)	12.5 (2.1; 39.6)	12.5 (0; 50)
	over 60 years (*n* = 13)	0 (0; 21.9)	10 (2.5; 60)	4.2 (0; 22.9)	0 (0; 25)
	P1*, P2*, P3*	ns., 0.0146, 0.0296	ns., ns., ns	ns., 0.0382, ns	ns., ns., ns

Distribution of AE-QoL domains (Function, Fatigue/Mood, Fears/Shame, Food); broken down by gender and age groups. The first number is the median, and numbers in brackets are the 25th and 75th percentiles, respectively

*AE-QoL* Angioedema Quality of Life questionnaire

P1 difference between 2016 and 2017; P2 difference between 2016 and 2018; P3 difference between 2017 and 2018

P1* difference between the 1st and 2nd age group; P2* difference between the 1st and 3rd age group; P3* difference between the 2nd and 3rd age group

##### Distribution of Domains Based on Gender

The scores of different questionnaire domains over the 3 years of the study were evaluated separately based on gender. Women reached a significantly higher score than men in the Function, Fears/Shame, and Food domains (*p* = 0.0032, *p* = 0.0021, *p* = 0.0040), while there was no significant difference in the Fatigue/Mood domain (Table 2).

##### Distribution of Domains Within Age Groups

Three years of follow-up were compared separately as well, based on the scores of different domains divided into age groups. The second age group reached the highest score in every domain (except the Fears/Shame domain in 2018) (Table 2).

#### Distribution of AE-QoL Total Scores and Domains Based on the Number of AE Attacks

There is a significant positive correlation between the AE-QoL total score and number of annual AE attacks in all 3 years of annual visits (2016–2018: *r* = 0.47; 2016: *r* = 0.43; 2017: *r* = 0.51; 2018 *r* = 0.47, *p* < 0.0001). Also, there was a positive correlation between the four domains and the total number of attacks in every year recorded. We further divided the population into two groups, based on the annual number of AE attacks. The first group included patient attacks per year under the median number, and the other group included patients whose number of attacks per year was over the median. The results show that both the total score and the domain score were higher in the group that had more than median AE attacks per year. There was a significant difference between the total score of the groups and the certain domains (Table 3)*.*

**Table 3:** Distribution of AE-QoL total scores and domains based on number of AE attacks

		AE-QoL	Function	Fatigue/Mood	Fears/Shame	Food
2016–2018	Under median attacks/year number (*n* = 153)	11.8 (0; 27.2)	0 (0; 28.1)	10 (0; 30)	8.3 (0; 25)	0 (0; 25)
	Above median AE attacks/year number (*n* = 133)	29.4 (14.7; 44.1)	31.3 (9.4; 50)	25 (15; 45)	25 (12.5; 47.9)	25 (0; 50)
2016	Under median AE attacks/year number (*n* = 53)	10.3 (0; 26.5)	0 (0; 25)	15 (0; 37.5)	8.3 (0; 25)	0 (0; 25)
	Above median AE attacks/year number (*n* = 42)	27.9 (16.9; 39)	31.3 (12.5; 50)	25 (8.8; 40)	25 (12.5; 45.8)	25 (0; 40.6)
2017	Under median AE attacks/year number (*n* = 53)	13.2 (2.2; 29.4)	6.3 (0; 37.5)	15 (0; 35)	8.3 (0; 25)	0 (0; 25)
	Above median AE attacks/year number (*n* = 44)	32.4 (15.4; 40.8)	28.1 (7.8; 48.4)	27.5 (15; 43.8)	27.1 (12.5; 41.7)	25 (0; 46.9)
2018	Under median AE attacks/year number (*n* = 47)	11.8 (0; 23.5)	0 (0; 25)	5 (0; 25)	8.3 (0; 25)	0 (0; 12.5)
	Above median AE attacks/year number (*n* = 47)	29.4 (13.2; 48.5)	31.3 (6.3; 50)	25 (10; 55)	20.8 (8.3; 62.5)	25 (0; 50)

Patients were divided into two groups: the first included patients for whom the attacks per year was *under the median*, and the second group included patients whose number of attack per year was *over the median*. The first number is the median, and the numbers in brackets are the 25th and 75th percentile values, respectively

*AE-QoL* Angioedema Quality of Life questionnaire, *AE* angioedema

#### Patients with Inverse Correlation Between AE-QoL Total Score and Number of AE Attacks

In the next part of the study, we chose 28 patients whose AE-QoL total score showed a negative correlation to the number of AE attacks (in 2016: *r* = −0.84, *p* = 0.0045; in 2017 *r* = −0.77, *p* = 0.0011, in 2018 *r* = −0.72, *p* = 0.0155); these patients were divided into two groups. The “A” group (*n* = 17) included patients who had AE-QoL score above the 75th percentile (> 36.76), but their number of AE attacks was below the median (< 5 attack/year). These patients had worse QoL despite having fewer attacks. The “B” group (*n *= 11) included patients who had the AE-QoL score under the median (< 22), but their number of attacks was above the 75th percentile (> 16 attack/year). These patients had a good QoL despite having more attacks.

Both groups showed a positive correlation between the Fatigue/Mood (“A” group *r* = 0.54, *p* = 0.0099; “B” group *r* = 0.72, *p* = 0.0048), Fears/Shame domain (“A” group: *r* = 0.55, *p* = 0.0077; “B” group: *r* = 0.54, *p* = 0.0467) and the total score. Group “B” had this correlation with the Food domain as well (*r* = 0.7, *p* = 0.0057).

#### Correlation Between the AE-QoL Total Score and Localization of Attacks

We evaluated the AE-QoL scores and attack location, based on the type of edema (subcutaneous of submucosal). Analysis was carried out for the 3-year follow-up and for each separate year. There was a positive correlation between attack locations and the total score.

#### Association Between the Domains and Localization

When evaluating the correlation between the domains and the localization, we found that the strongest correlation was between the localization (both types) and the Function domain.

#### Correlation Between the AE-QoL Total Score and Complement Parameters

The total score received after the evaluation of the AE-QoL questionnaires was compared with complement laboratory parameters obtained in each visit. No significant correlation was found between the AE-QoL total score and C4 (*r* = −0.08; *p* = 0.2029), C1-INH concentration levels (*r* = −0.18; *p* = 0.0026) and C1-INH functional activity (*r* = −0.07, *p* = 0.2133). This result was confirmed by annual comparisons as well. Out of the complement parameters, C4 (*p* < 0.0001; Spearman *r* = −0.26) and the functional activity of C1-INH (*p* = 0.0002; Spearman *r *= −0.22) showed a negative correlation to the annual number of AE attacks.

#### AE-QoL Data From Three Subsequent Years

Out of 125 patients, 61 completed AE-QoL questionnaire in three subsequent years. We studied how the AE-QoL total scores of these patients have changed during certain study years. A change of ± 6-point difference was selected to be an index of worsening or an improving. Among patients whose QoL has worsened in 2017 (a higher AE-QoL score was reached, *n* = 16), the Fear/Shame (16/16) and Fatigue/Mood (15/16) domains have changed in most patients. In patients whose QoL improved (18), the Fear/Shame (17/18) and the Functioning domains (17/18) have changed. There was no change in the type and dose of medications used for long-term prophylaxis during the observational period. (Out of those 16 patients whose QoL has decreased, and out of those 18 whose QoL has improved, 7–7 have received a long-term prophylactic treatment.

### Patients with Acquired Angioedema (C1-INH-AAE)

In 10 C1-INH-AAE patients (age: median 59 years, range 49–83), 6 out of 10 patients had some kind of hematologic disorder as well. The median AE-QoL total score was 30.9, and the median of number of AE attacks was 0.5. From the domains of the questionnaire, the Fatigue/Mood and Fear/Shame domains received the highest score (median 50 and 37.5, respectively). This was followed by Food (25) and Functioning (6.3). There was a significant correlation between the annual number of attacks and the AE-QoL total scores (*r* = 0.46; *p* < 0.0001). Similar to C1-INH-HAE, AE-QoL and complement values were not correlated.

**Fig. 1:**
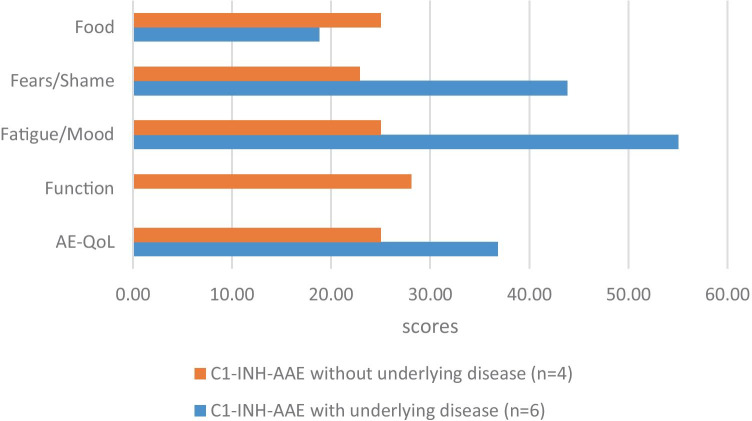
AE-QoL total and domain scores of C1-INH-AAE patients. The AE-QoL total and domain scores were compared between patients with acquired angioedema (C1-INH-AAE) without and with underlying disease. C1-INH-AAE patients who had an underlying disease reported worse QoL, and the Fatigue/Mood and “Fear/Shame” domain scores were worse as well. The “Function” domain score was 0 in patients with C1-INH-AAE and underlying disease (s). C1-INH-AAE: acquired angioedema with C1-inhibitor deficiency

Those C1-INH-AAE patients who had an underlying hematologic/lymphoproliferative disease reported worse QoL, and the Fatigue/Mood and Fear/Shame domain scores were worse as well. In contrast, Functioning and Food domains were reported worse for those who did not have any kind of underlying disease (Fig. 1).

## Discussion

The AE-QoL questionnaire was proven reliable and is a validated tool for the assessment of patients with different types of AEs, such as Histamine-mediated angioedema, or BK-mediated hereditary angioedema [18]. The original AE-QoL applies to a period of 4 weeks preceding the visit, and provides information on the QoL of the patients; therefore, it can be utilized in clinical trials where patients have frequent follow-up visits. In contrast, in routine clinical practice, patients are usually monitored annually. Our study is the first survey to use the AE-QoL questionnaire during the annual follow-up visits of patients with both C1-INH-HAE and C1-INH-AAE. Additionally, this study analyzes a 3-year period, comparing AE-QoL results with the clinical features and laboratory parameters yearly.

Weller et al. found a positive correlation between the number of attacks occurring in the 4 weeks preceding the completion of the questionnaire and the total score of the AE-QoL [20]. To our surprise, we found that the AE-QoL total score and number of attacks per year in our study design also correlated, but we are aware that a recall bias may occur when AE-QoL data of a whole year is analyzed. It is important to emphasize that the QoL of a patient can also change year by year. Considering this and the fact that there is a great variety in the natural course of the disease, the QoL of the individual patient needs a regular revision, for which annual follow-up visits are a good option.

When AE-QoL results were analyzed by age groups, the highest scores were reached by the 41–60 age group. Also, the 18–40 and 41–60 age groups received the highest score by domains, thus presenting the worst QoL. We therefore assume that active, working-age group patients are the most sensitive for QoL changes. We also observed that women reached a higher AE-QoL total score than men in all 3 years of observation; however, female patients over 60 reached a lower overall score than younger women; the latter tendency was not observed in men. We can speculate that such disparity is caused by menopause, during which the levels of estrogen decrease. This sex hormone influences the production of BK, which is the main vasoactive mediator causing AE attack [25–27].

In contrast, no correlation was found between AE-QoL total score and complement parameters. Therefore, monitoring complement parameters is not helpful regarding the follow-up of patients’ QoL.

A fundamental question may rise: if the AE-QoL score correlates so well with the number of AE attacks, would it be sufficient to assess the severity of the disease exclusively based on the number of AE attacks? We believe that number of attacks is not a sufficient parameter. In our study, we found a smaller group of patients that show an inverse correlation between the AE-QoL total score and number of AE attacks: part of this group has a worse QoL despite a low number of AE attacks, while others have many attacks, but their QoL were considered to be good. In the inverse correlation patient group, the total AE-QoL score correlated best with two domains, namely, the Fatigue/Mood and Fears/Shame domains, which reflects the observations that anxiety and depression occur more frequently in patients with C1-INH-HAE than in the average population [14, 15, 28]. This differs significantly from the results of those patients’ AE-QoL questionnaires whose AE-QoL total score positively correlated with the number of AE attacks. For these patients, the Functioning domain correlated with the AE-QoL total score.

Another pertinent question is if the AE-QoL could help in developing a therapeutic strategy, and most importantly if it might be helpful in assessing the introduction of long-term prophylaxis. We suggest that a long-term prophylactic treatment can be initiated after considering the AE-QoL score, even in patients with worse QoL despite having few attacks. Initiating a prophylactic medication is expected to increase the patient’s sense of security and, later, will presumably affect their QoL as well. On the other hand, for patients who assessed their QoL as “good” despite having frequent attacks, the introduction of a long-term medication treatment is worth discussing, since reducing the number of AE attacks also reduces the possibility of potentially life-threatening conditions.

Additionally, the specific evaluation of certain domains of the AE-QoL can help in the decision to apply further supplementary treatments. These may include supportive daily-life coaching, consultation with a psychologist, psychotherapy, and starting of a mood improving treatments.

Another novel aspect of this study was the application of the AE-QoL questionnaire for C1-INH-AAE patients. This unique patient group showed a higher score in the Fatigue/Mood and Fears/Shame domains and demonstrated a positive correlation to the number of AE attacks per year. As expected, C1-INH-AAE patients which in most cases have an underlying disease showed a higher total QoL score, possibly due both to HAE and to underlying conditions. Other conclusions should be cautiously drawn in view of the small number of patients, and because the questionnaire has only been completed once. We plan to follow up these patients during their annual visits with the AE-QoL questionnaire.

The study’s major limitation is that we used a questionnaire that was validated for a 4-week recall to correlate with 1 year’s data. In the future, it would be worth considering a validation of QoL questionnaires with a larger scope (i.e., last 1 year), or compare the results of questionnaire based on the last 4 weeks with a full-year QoL instrument.

## Conclusions

Based on our results, we can conclude that the AE-QoL is a useful tool and can be used during annual visits in the clinical practice. The AE-QoL total score is a good indicator of disease activity both in C1-INH-HAE and in C1-INH-AAE. The comprehensive analysis of the questionnaire may help in developing an individualized therapeutic strategy, and in devising individual mental/psychologic management and other complementary therapies.

## Data Availability

Semmelweis University, Hungarian Angioedema Center of Reference and Excellence.

## Abbreviations

AAS: Angioedema Activity Score
AE: Angioedema
AE-QoL: Angioedema Quality of Life Questionnaire
BK: Bradykinin
C1q, C3, C4: Complement system components
C1-INH: C1 inhibitor
nC1-INH-HAE: Hereditary angioedema with normal C1-inhibitor deficiency
C1-INH-AAE: Acquired angioedema with C1-inhibitor deficiency
C1-INH-HAE: Hereditary angioedema with C1-inhibitor deficiency
CH50: Total complement activity
DLQI: Dermatology Life Quality Indexet
HACRE: Hungarian Angioedema Center of Reference and Excellence
HAE-QoL: Hereditary angioedema Quality of Life
HR-QoL: Health-related quality of life
SF-36: Short Form 36 Health Survey
QoL: Quality of Life

## Code Availability

GraphPad Prism 5.0 (GraphPad Software, San Diego, California, USA).
